# Supplementary material for: Dietary Counseling Outcomes in Patients with Lung Cancer in an Upper-Middle-Income Country: An Open-Label Randomized Controlled Trial
Source: J Clin Med. 2024 Sep 4;13(17):5236. doi: 10.3390/jcm13175236 (PMC11396147; doi:10.3390/jcm13175236)
Supplement: Supplementary file 1 [file jcm-13-05236-s001.zip › jcm-3154758-supplementary.pdf]

Supplementary Figures

Dietary Counseling Outcomes in Patients with Lung Cancer in an Upper-Middle-Income Country: An Open-Label Randomized Controlled Trial

Busyamas Chewaskulyong [1,*], Haritchanan Malairungsakul [2], Supawan Buranapin [3], Panas Jesadaporn [4], Thanika Ketpueak [1], Thatthan Suksombooncharoen [1] and Chaiyut Charoentum [1]

[1] Division of Medical Oncology, Department of Internal Medicine, Faculty of Medicine, Chiang Mai University, Chiang Mai 50200, Thailand; thanika.k@cmu.ac.th (T.K.); thatthan.s@cmu.ac.th (T.S.); chaiyut.charoentum@cmu.ac.th (C.C.)
[2] Division of Gastroenterology, Department of Internal Medicine, Faculty of Medicine, Chiang Mai
University, Chiang Mai 50200, Thailand; haritchm@gmail.com
[3] Division of Endocrinology and Metabolism, Department of Internal Medicine, Faculty of Medicine,
Chiang Mai University, Chiang Mai 50200, Thailand; supawan.b@cmu.ac.th
[4] Division of Geriatric Medicine, Department of Internal Medicine, Faculty of Medicine, Chiang Mai University, Chiang Mai 50200, Thailand; panas.j@cmu.ac.th
* Correspondence: bchewask@gmail.com; Tel.: +66-53935482-5

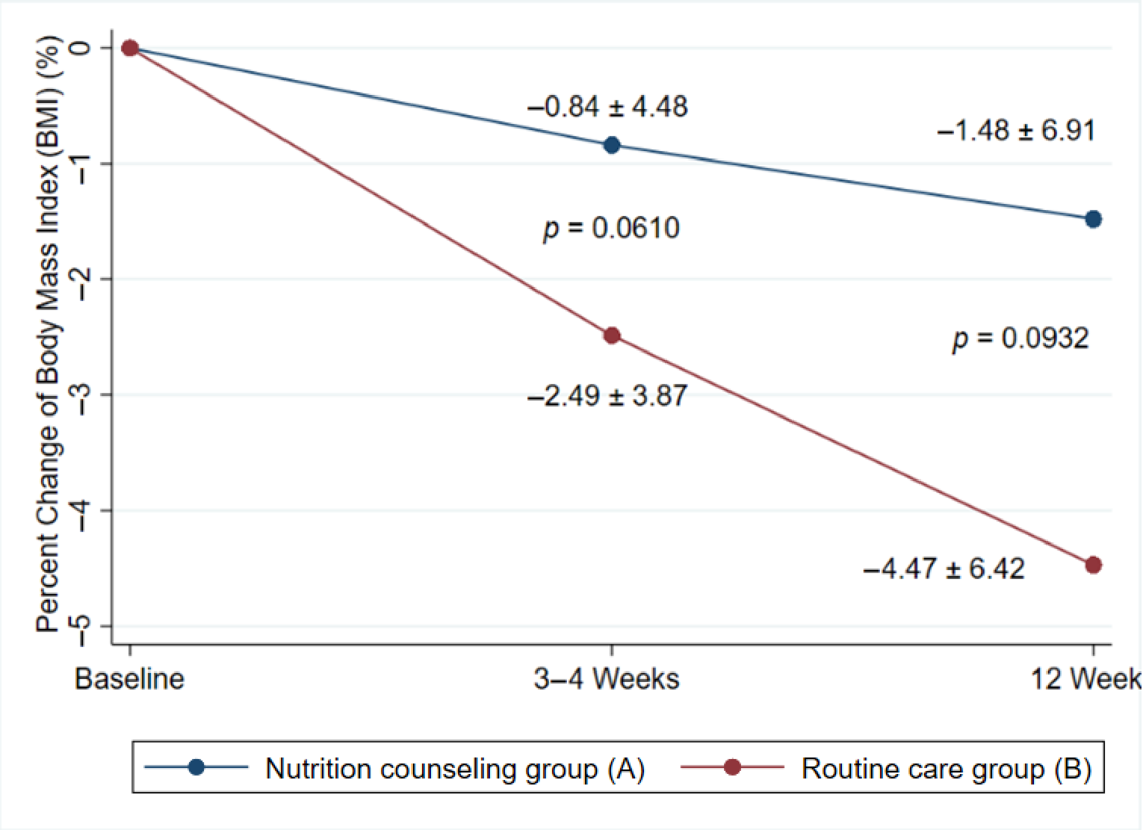

Figure S1: Percent Change of Body Mass Index (BMI).

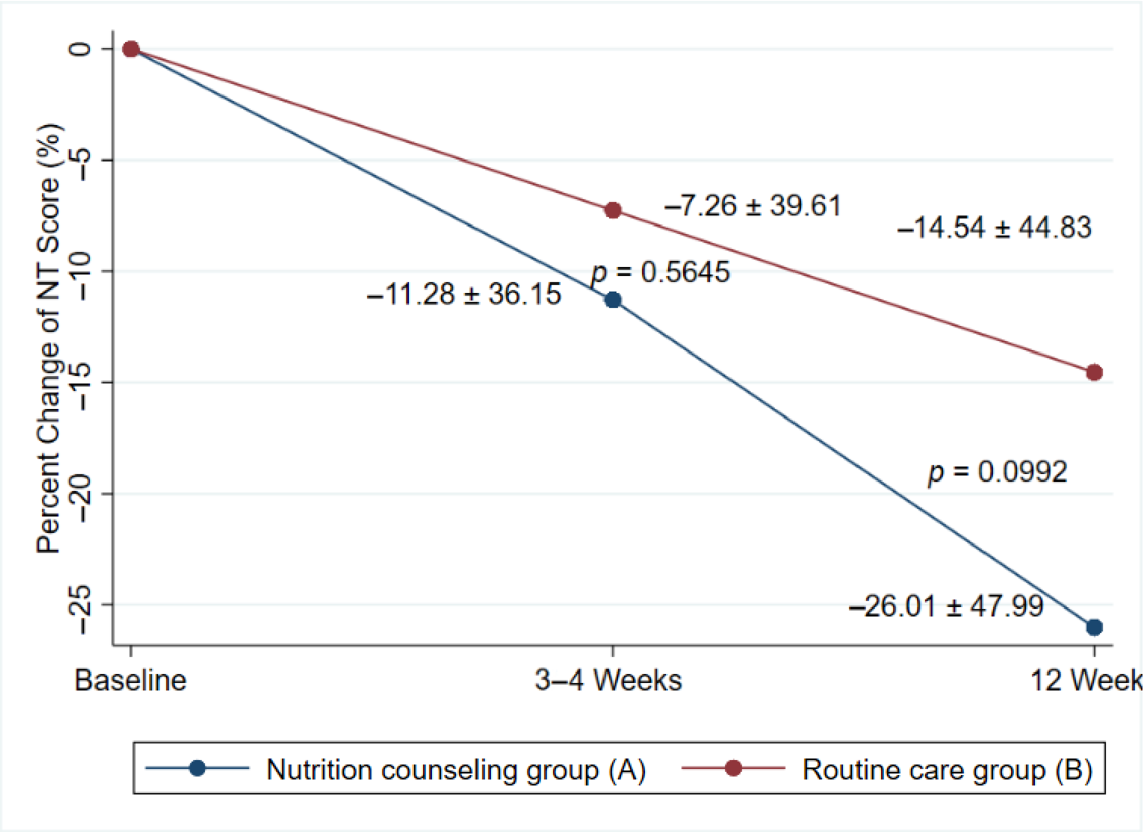

Figure S2: Percent Change of Nutrition Score.

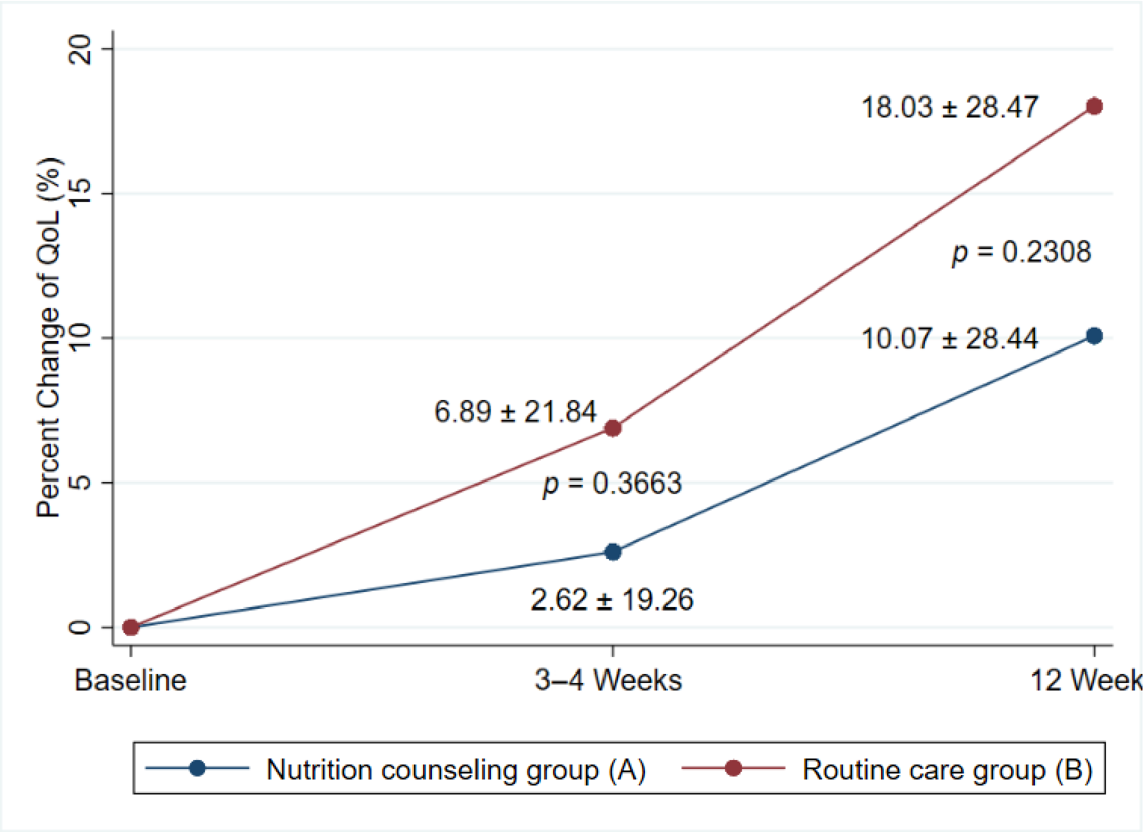

Figure S3: Percent Change of Quality of Life.

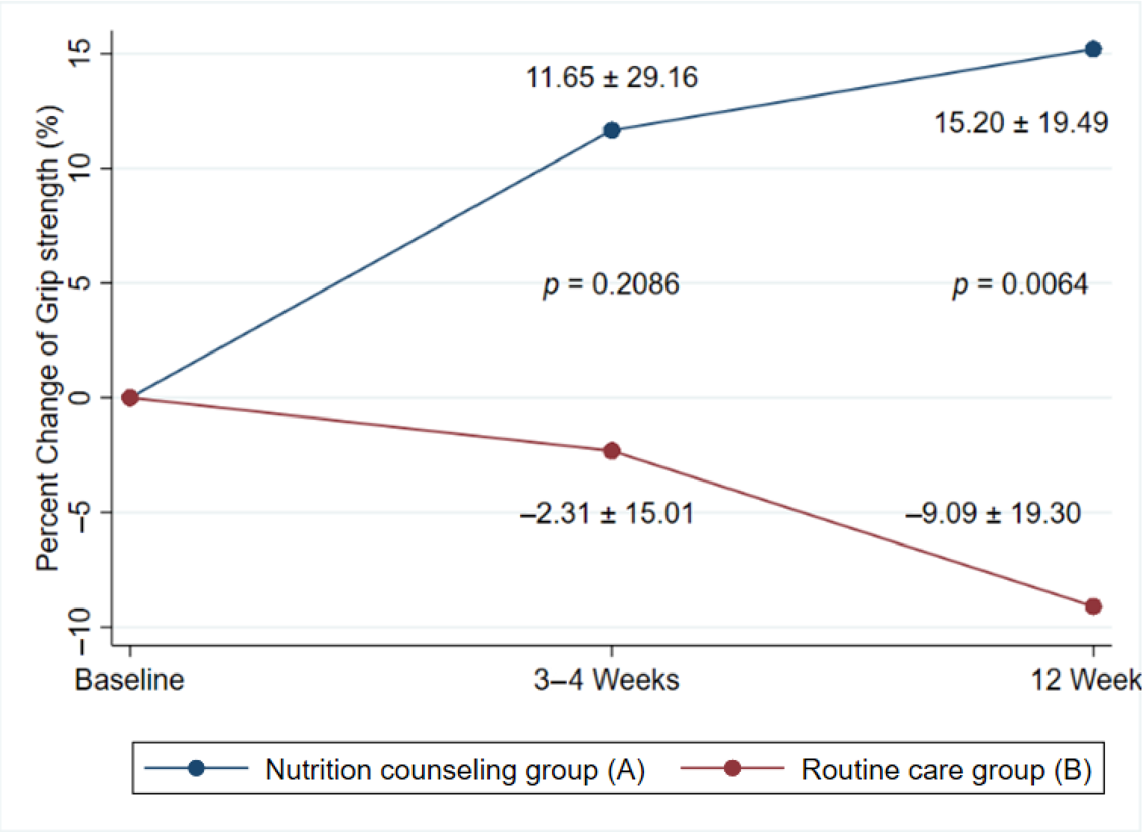

Figure S4: Percent Change of Grip Strength.

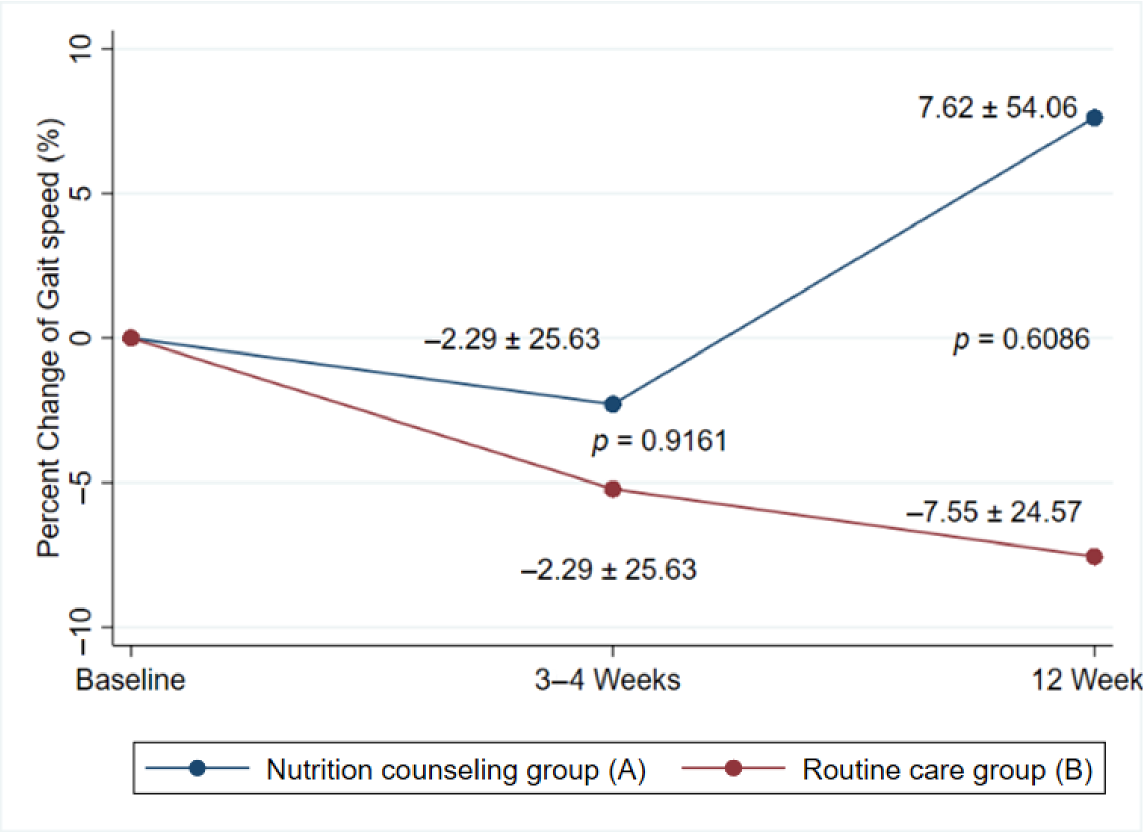

Figure S5: Percent Change of Gait Speed.

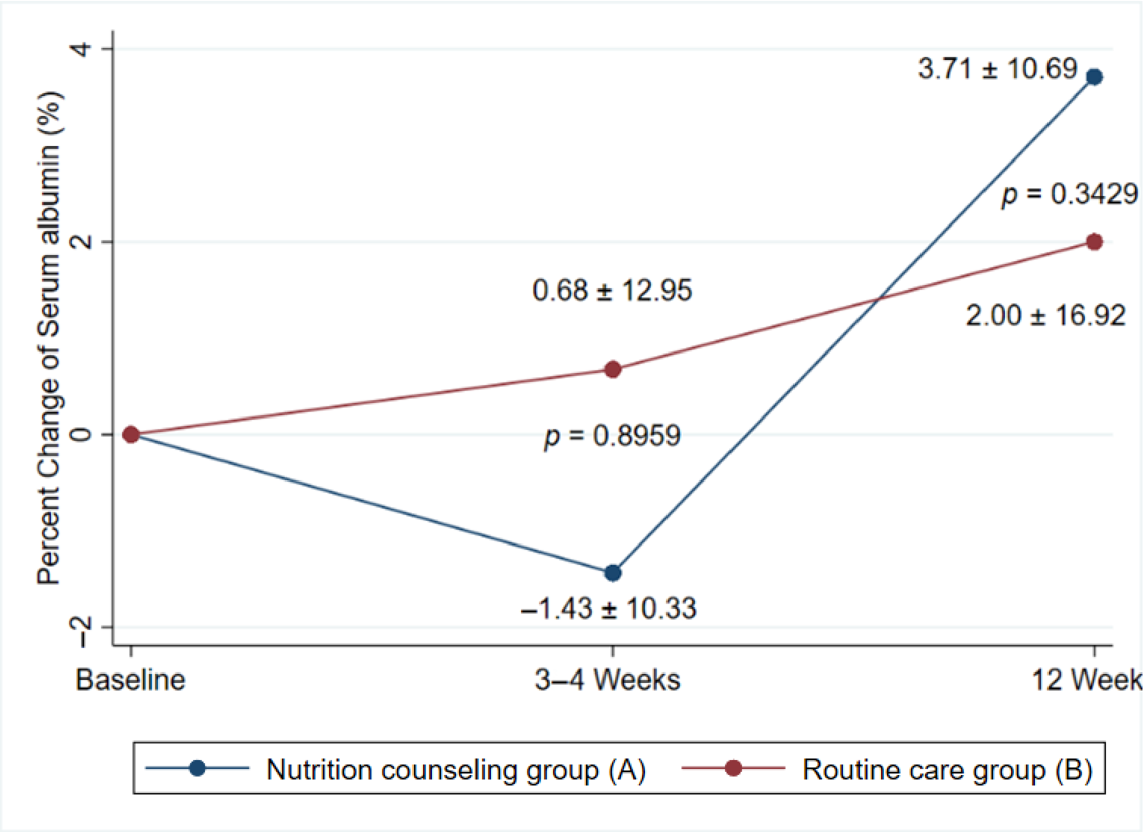

Figure S6: Percent Change of Serum Albumin.

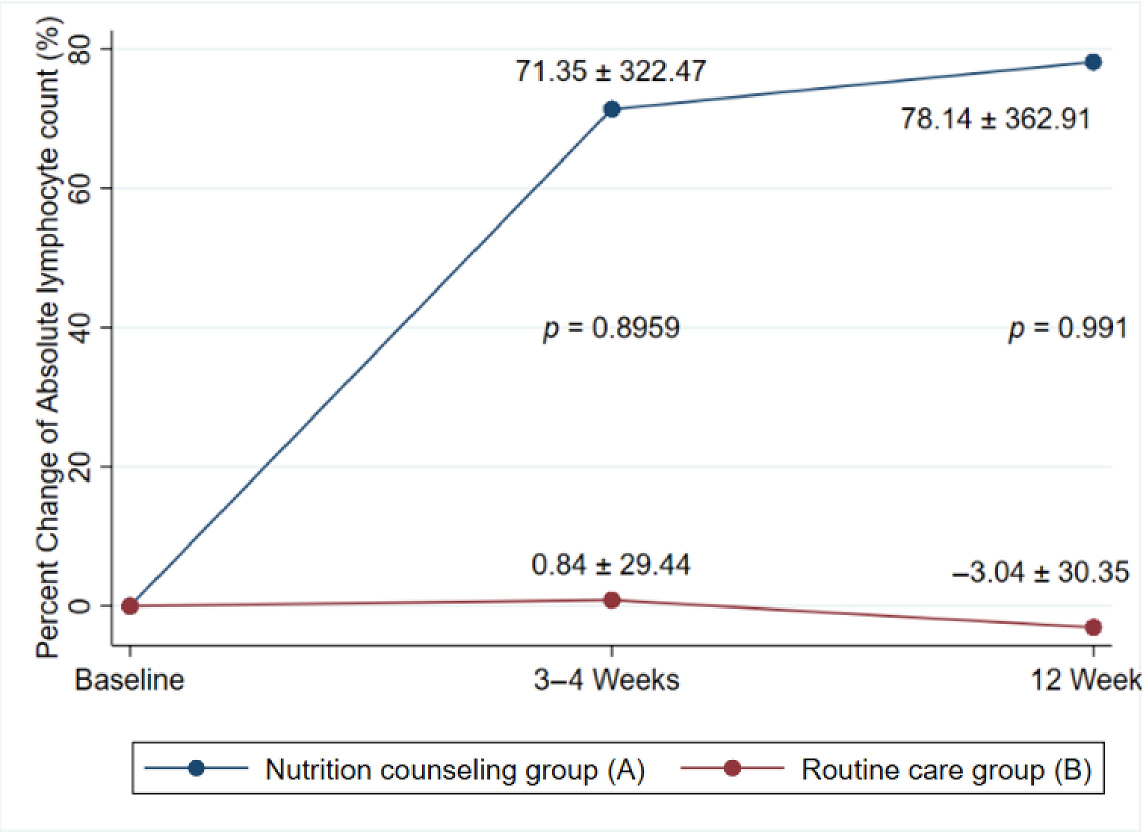

Figure S7: Percent Change of Absolute Lymphocyte Count.

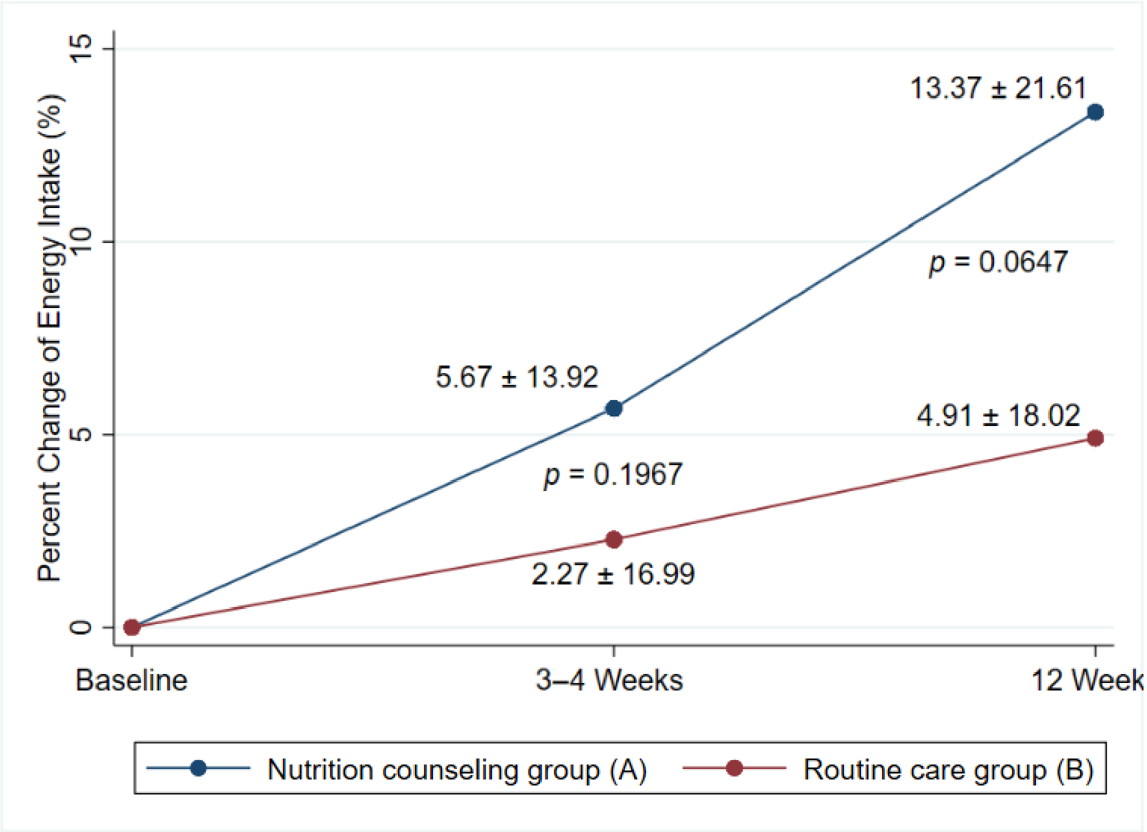

Figure S8: Percent Change of Energy Intake.

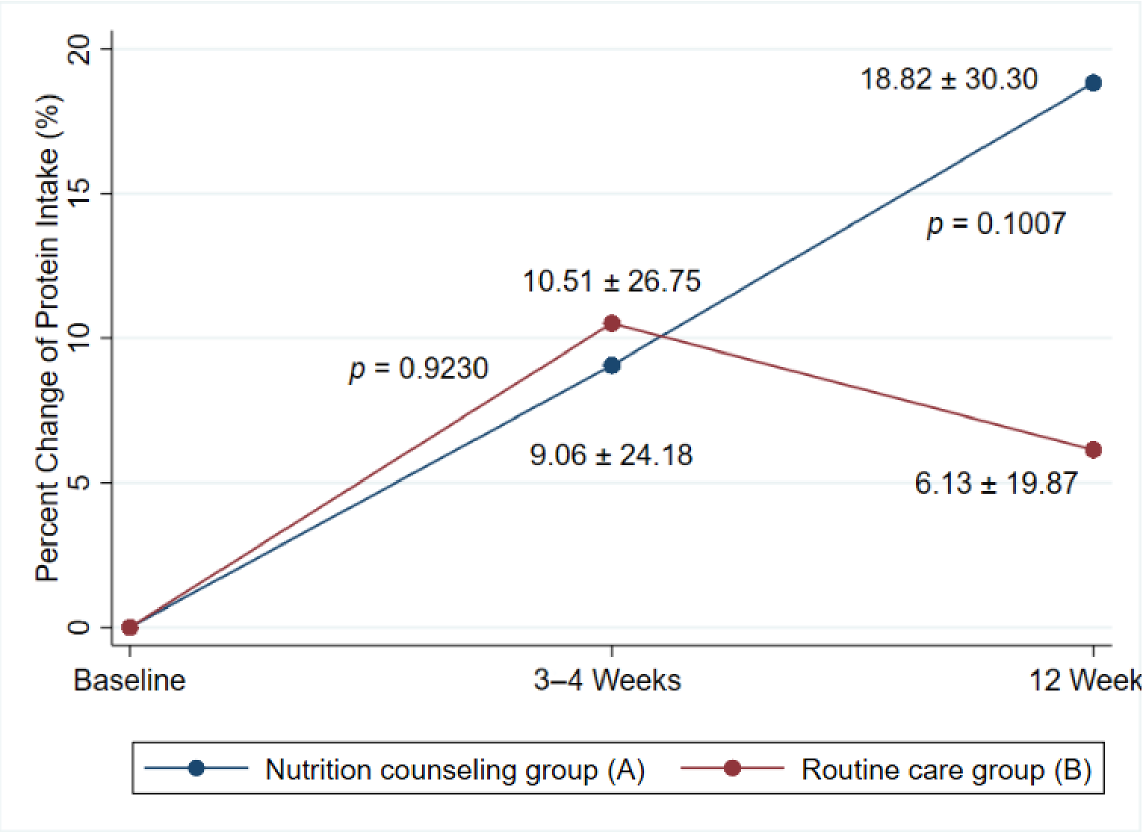

Figure S9: Percent Change of Protein Intake.

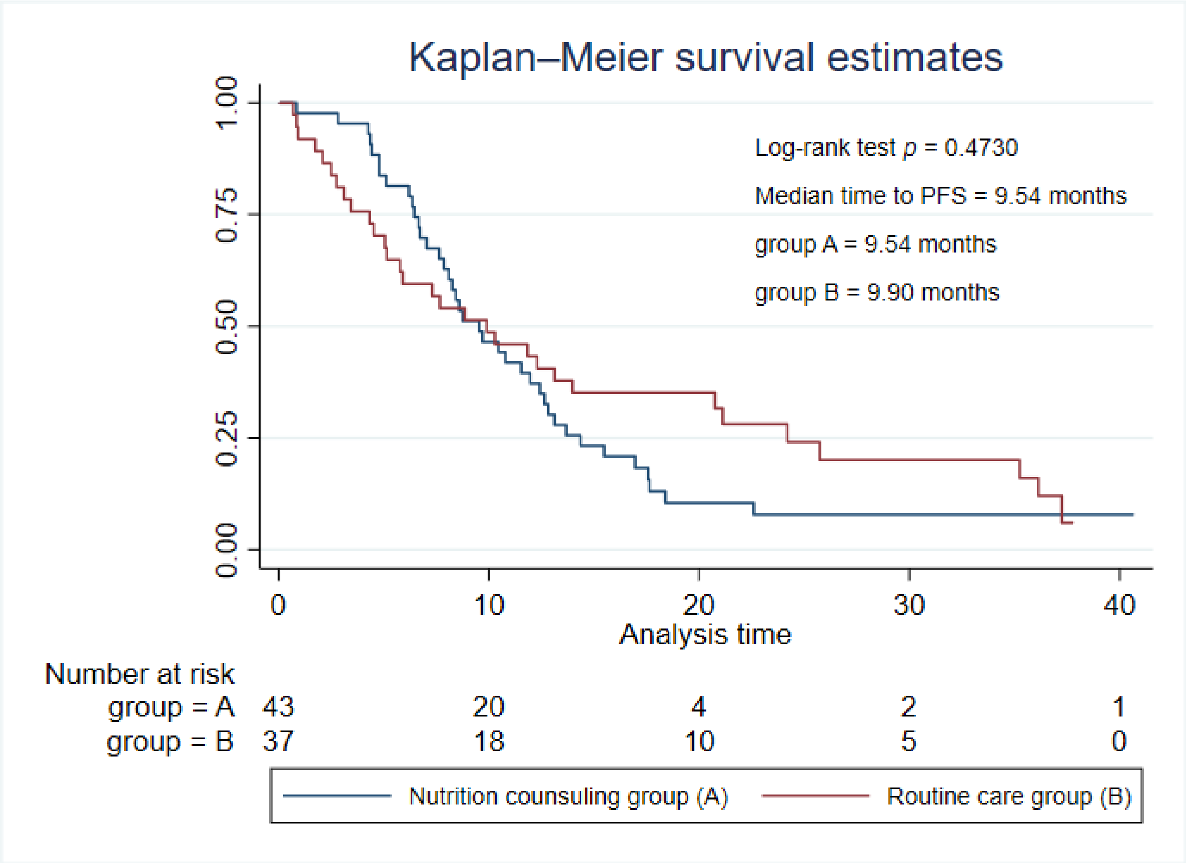

Figure S10: Progression-free Survival.

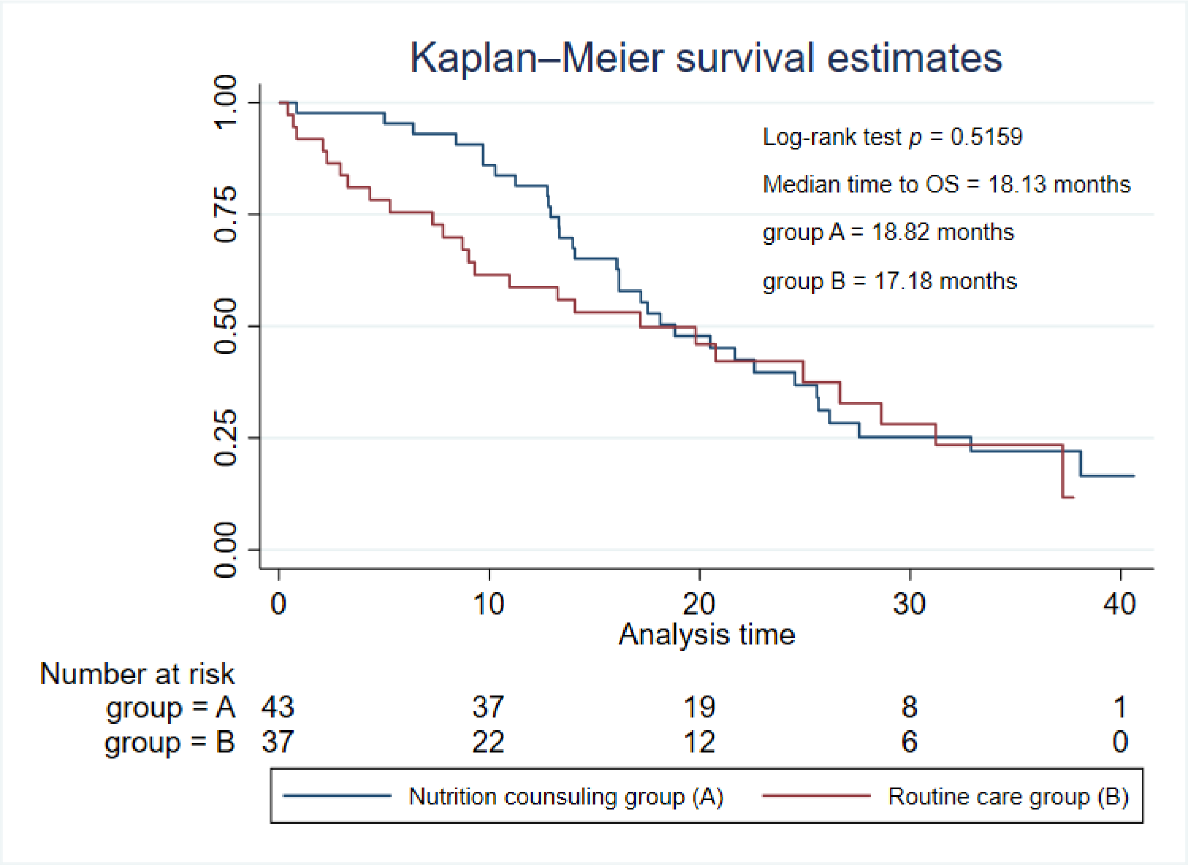

Figure S11: Overall Survival.
